# Investigating the Role of Mucin as Frontline Defense of Mucosal Surfaces against *Mycobacterium avium* Subsp. *hominissuis*

**DOI:** 10.1155/2020/9451591

**Published:** 2020-06-29

**Authors:** Jessica Bechler, Luiz E. Bermudez

**Affiliations:** ^1^Department of Biomedical Sciences, Carlson College of Veterinary Medicine, 106 Dryden Hall Corvallis, Corvallis, OR 97331, USA; ^2^Department of Microbiology, College of Science, Oregon State University, Corvallis, USA

## Abstract

*Mycobacterium avium* is a human and animal pathogen that infects the host through the mucosal surfaces. Past work has demonstrated that the bacterium can interact with both the respiratory and gastrointestinal tracts. Those surfaces in the body are covered by a bilayer of a glycoprotein, mucin, which works as a physical barrier and a gel which contains antibacterial and antivirus properties. This current work shows that different strains of *M*. *avium*, in contrast to *Escherichia coli*, *Pseudomonas aeruginosa*, and *Listeria monocytogenes*, are not able to bind to mucins, MUC2 and MUC5b, the main mucins in the gastrointestinal and respiratory tracts, respectively. The lack of binding is due to the characteristics of the cell wall and is impaired by altering lipids, proteins, or glycolipids. *M*. *avium*, in contrast to *E*. *coli*, interacts with epithelial cells equally in the presence or absence of the mucin, suggesting that the cell wall of the pathogen can facilitate the bacterial movement through the mucin layer, towards the mucosal wall. In conclusion, the study has shown that *M*. *avium* can avoid the mucin barrier, which explains its ability to interact with the mucosal epithelium, even in absence of motion-related structures.

## 1. Introduction

*Mycobacteriumavium* subsp. *hominissuis* (*M*. *avium*) is an environmental bacteria associated with the infection of individuals with underlying lung conditions such as bronchiectasis, cystic fibrosis, and emphysema [1, 2]. Infections caused by *M*. *avium* are also seen in patients with immunosuppression, such as AIDS and specific immunodeficiencies [3].

*M.avium* interacts with the host mucosal cells once in the airways or in the GI tract. Epithelial mucosal cells are invaded by the pathogen both in vitro [4] and in vivo [5] as a step toward the infection. *M*. *avium* pathogenesis of the lung is also associated with the formation of a biofilm on the surface of the mucosa [6, 7]. This infection is complex and involves the formation of microaggregates once they bind to the epithelial mucosa and stimulation of cytoskeleton rearrangement, stages that require the participation of many bacterial proteins [8, 9].

Healthy airway surfaces are coated with a mucus layer that is largely composed of water, salts, mucins, and surfactants. As a step in the process of interaction with the airway or the GI mucosa, pathogens need to bypass the mucin layer, both in the respiratory tract as well as the intestinal tract. In the intestinal lumen, mucin is present in two layers, one adjacent to the mucosal surface, which is rich in antimicrobial peptides, and a more external layer, where bacteria are encountered [10, 11]. The same arrangement appears to be present in the airways [12].

Mucins are high-molecular mass glycoproteins composed of a protein core with carbohydrate and sulfate molecules. Mucins have characteristic Pro-Thr-Ser repeats as well as a Cys-rich domain that provide key disulfide bonding capacity, important for mucin multimerization and mucus function. The major mucins produced in the airways are the secreted polymeric mucins MUC5AC and MUC5B [13], while in the GI mucosal surface, MUC2 is more abundant [11]. In mammalians, the mucin/mucus involves the coordinated activities of secretory cells that release polymeric mucin glycoproteins and ciliated cells that transports and eliminates foreign material including bacteria [13]. Bergstrom et al. and colleagues [14] showed that intestinal MUC2 can protect against lethal infectious colitis by disassociating pathogenic and commensal bacteria from the colonic mucosa. The authors showed that MUC2 production is critical for host protection during attachment and effacing bacterial infections (*Escherichia coli* and *Citrobacter rodentium*) but limits the overall bacterial pathogens and commensal numbers associated with the mucosal surface. Mucins also reduce surface adhesion and biofilm formation of *Pseudomonas aeruginosa* [15].

In addition, the mucin interacts with pulmonary macrophages (alveolar and submucosal) in several capacities in distinct anatomic locations. The viscosity of the environment caused by the glycoprotein mucin is very important to keep pathogens at some distance from the mucosal surface. For some pathogens, motility plays an important role in pathogenesis and is crucial to organisms such as *Pseudomonas aeruginosa* to colonize the host and form biofilms [16].

The hallmark phenotype of cystic fibrosis is dehydration of the mucosal layer across all the epithelial surfaces in the body.

The abilities of secreted mucins to regulate mucocellular clearances are dependent on their polymer structures formed through disulfide bonds. Pathogenic bacteria such as enterotoxic *Escherichia coli* produces a protein, EatA, that degrades the intestinal mucin [17], and therefore can migrate towards and ultimately bind to the mucosal surface.

Because the role of mucins has not been studied with regards to *M*. *avium* infection, we hypothesized that the mucin is probably ineffective in its role to separate mycobacteria from the mucosal layer of cells, likely due to the lipid content of the mycobacterial surface, therefore facilitating the bacterium movements toward the mucosal wall. The results of the present investigation support the hypothesis that the *M*. *avium* surface protects the bacterium against the action of mucins.

## 2. Materials and Methods

### 2.1. Bacteria

*M*. *avium* 104 was isolated from the blood of a patient (the characteristics of the bacteria have been published in [18]). Bacteria were cultured in the Middlebrook 7H10 agar medium (Difco Laboratories, Detroit, MI), supplemented with oleic acid, albumin, dextrose, and catalase (OADC). *M*. *avium* 3388 obtained from Barbara Brown (Taylor, Texas) was cultured in the Middlebrook 7H10 agar medium described above. It was a lung pathogen. *Mycobacterium avium* (MAH) A5 and MAH A5 GPL/4B2 mutant were previously described [18, 19].

*Pseudomonas aeruginosa* PD0300 mucA22 mucoid variant was kindly provided by Martin Schuster (Oregon State University). *P*. *aeruginosa* was grown in the Mueller–Hinton broth. Bacteria were washed (3,000 ×g), and the inoculum was established in the phosphate buffer solution (PBS) and inoculated in the 7H9 broth. *Escherichia coli* ATCC CFT073, *Staphylococcus aureus* ATCC 25923, and *Listeria monocytogenes* (clinical isolate) were cultured on the Luria–Bertani (LB) agar.

Prior to performing the assays, dilutions were made in Hank's balanced salt solution (HBSS) to match McFarland standards. Bacteria were prepared at 10^5^, 10^6^, and 10^7^ concentrations.

### 2.2. Mucin

Purified gastric mucin MUC2 and pulmonary MUC5b purchased from Pfaltz and Bauer Inc. were added to a 2% agar gel at 5% *w*/*v*. Agar 2% *w*/*v* was used as the control. Twenty four well tissue culture plates were used with approximately 400 *μ*L in each well for the binding assays.

### 2.3. Binding to Mucin Assay

**Figure 1:**
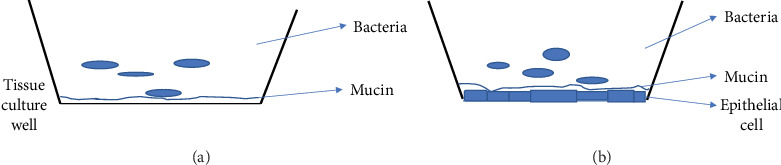
Models used to determine the binding of bacteria to the mucin and epithelial cell invasion: (a) polypropylene well and (b) epithelial cells covered with a layer of mucin.

Bacteria inoculum prepared in HBSS at the pH 7.3 was diluted to a concentration of approximately 6 × 10^3^ CFU/ml as determined by comparison with the McFarland standard. Then, 200 *μ*L of the suspension was added to each tissue culture plate well. The plate containing the agar, agar plus mucin, and the final bacterial suspension was incubated for 3 h at 37°C under anaerobic conditions (anaerobic jar, BD BBL GasPak jar). After the incubation period, each well was washed three times with 300 *μ*L of HBSS, and the removed supernatant was collected in 5 mL plastic tubes. Then, it was diluted by 10^−3^, and the final suspensions were plated onto the 7H10 agar or LB agar plates. The number of CFU on the plates was determined after 10 days for mycobacteria and 24–48 hours for other bacteria (Figure 1).

### 2.4. Pretreatment of Bacteria

To evaluate a variety of environmental conditions, usually encountered in the process of infection that could impact *M*. *avium* binding to the mucin, bacteria were preincubated under different conditions, and the mucin attachment experiments were repeated. *M*. *avium* is encountered in diverse environmental conditions, such as water. In addition, *M*. *avium* before reaching the intestinal tract has to pass through the stomach (2 h emptying time). The pH of the stomach was considered to be 3.4, following ingestion of food. After incubation under the environmental condition, bacteria were spun down to 4°C, and the concentration of the bacteria on the suspension was determined by the turbidity [20].

To determine the role of lipids and proteins on the bacterial surface during binding, mycobacteria were incubated with either Tween-20 for 3 h at 37°C or 50% trypsin for 3 h at 37°C. Following treatment, bacteria were spun down and the final inoculum was prepared at 4°C to prevent the change of the bacterial phenotype as described above.

To evaluate whether the bacterial surface was involved in the property of not binding to the mucin, we use the 4B2 mutant, which contains a mutation in the gene *pstB* involved in GPL synthesis, a mutant that has an altered colony morphotype [19].

### 2.5. Invasion Assay

Laryngeal cell line HEp-2, purchased from ATCC (American Tissue Culture Collection), was maintained in RPMI-1640 supplemented with 10% fetal bovine serum (FBS). Lung alveolar epithelial cells (A549) obtained from ATCC were cultured in DMEM supplemented with 10% FBS. Monolayers were prepared by seeding 10^5^ cells in each well of a 24-well tissue culture plate and incubated until achieving 90% confluency. Monolayers were then covered with 5% mucin MUC2 or MUC5b, and the ability of bacteria to enter the epithelial cells was investigated as previously reported [8, 9]. In brief, monolayers were incubated with either *M*. *avium* 104 or *P*. *aeruginosa*, *E*. *coli*, *S*. *aureus*, and *L*. *monocytogenes* for 1 hour, the supernatant was removed, and the plate was washed with HBSS three times. The epithelial cell monolayer was than incubated at 37°C for 4 hours. Following the incubation, monolayers were lysed as previously described [8, 9], and the lysate was serially diluted and plated into 7H10 agar plates (Figure 1).

In some of the assays, bacteria were treated with 4 *μ*g/ml of polymyxin B (antimicrobial peptide surrogate) alone or incubated with polymyxin B MUC2 mucin on a HEp-2 cell monolayer.

### 2.6. Statistical Analysis

The data were analyzed using GraphPad Prism. Student's *t*-test and ANOVA were used to determine the significance of the comparisons.

## 3. Results

### 3.1. *M*. *avium* Mucin Adhesion

To determine whether *M*. *avium* strains can bind to the mucin that comprises the intestinal mucosa, bacteria were incubated using an inoculum of approximately 10^8^ CFU/ml bacteria on the MUC2 mucin-covered tissue culture plate. After the incubation, each well in the mucin-covered plate was washed three times at room temperature with PBS. The bacteria removed was plated and counted as the nonadhered bacteria. A similar assay was carried out using mucin MUC5b.

*Pseudomonas aeruginosa* PD0300, a mucA22 mutant of PAO1, was used as a positive control for this assay as it binds to various mucins, including the porcine stomach mucin used as previously established [21, 22]. With the basic protocol, PD0300 showed a significant amount of binding to the mucin/agar compared with agar, whereas *M*. *avium* 104 showed little to no significant amount of binding to the mucin/agar compared with agar.

To determine whether binding to mucins was related to the pathogenic bacterium being Gram positive or Gram negative, *Listeria monocytogenes*, *Staphylococcus aureus 25923*, *Escherichia coli* CFT073, and *Mycobacterium avium* strains *104 and 3388* were assayed for mucin MUC2 binding. Gram-negative bacterial species (*E*. *coli* and *P*. *aeruginosa*) and *Listeria monocytogenes* showed a statistically significant greater percent of binding to the mucin/agar compared to agar, and both *S*. *aureus* and *M*. *avium* showed little to no significant binding to the mucin/agar compared to agar (Table 1). In all *M*. *avium* species assayed, there was no significant difference seen in binding to the mucin/agar compared to agar. The results obtained regarding the binding to the MUC5b mucin were very similar (Table 2).

### 3.2. Effect of Environmental Condition on Binding to Mucin

To further investigate the possible factors affecting the binding of *M*. *avium* 104 to the MUC2 mucin, a number of pretreatments were performed prior to the binding assay basic protocol. Pretreatments of bacteria with DI H_2_O and HCl (pH 3.4 and H_2_O) were done directly prior to the assay; *M*. *avium* strains (104 and 3388) were incubated in water (12 h) or acid with a pH of 4 (2 h), and the ability to bind to the mucin was examined. As shown in Table 3, environmental condition incubation had no effect on the binding to the mucin.

### 3.3. Adherence to Mucosal Epithelial Cell

*M*. *avium* does not express a flagellum, and therefore does not move quickly through the mucus layer. To determine whether a layer of mucus would interfere with the ability of *M*. *avium* to bind and invade mucosal epithelial cells, invasion of HEp-2 and A549 alveolar epithelial cells was evaluated in the presence or absence of a MUC2 mucin layer. As shown in Table 4, although the mucin interferes with the binding and invasion of host cells by *E*. *coli*, it had no effect on the invasion of the mucosal cells by two different strains of *M*. *avium*.

### 3.4. Treatment of *M*. *avium* with Tween-20 or Trypsin

The ability to prevent the binding to the mucin can be related to the cell wall of *M*. *avium*. To investigate the role of the bacterial surface, we treated *M*. *avium* with 5% Tween-20 and 50% trypsin, prior to determination of binding to the mucin. Both treatments significantly increased the binding of *M*. *avium* to the mucin, suggesting that the pathogen is surrounded by a cell wall that prevents the binding to the mucin and removal of the mucosal surface by a mechanical mechanism (Table 5).

The indication from the previous results suggests that the bacterium cell contents are very likely involved in the ability to prevent interaction with the mucin. To confirm the role of the bacterial surface on the binding to the mucin, we employed the *M*. *avium* 4B2 mutant (mutation on the gene *pstB*, a synthase part of the glycopepdolipid synthesis pathway, 34) in the mucin binding assay. The results show that the *M*. *avium* 4B2 mutant can bind to the mucin, in contrast to the wild-type bacteria (Table 6).

### 3.5. Effect of Polymyxin B on *M*. *avium* and Invasion of HEp-2 Cells

To evaluate whether polymyxin B, a surrogate for the antimicrobial peptide, had an antibacterial effect on a system containing mucosal cells and MUC2 mucin, both *M*. *avium* 104 and MAH mutant 4B2 were incubated with 4 *μ*g of polymyxin B for 4 hours, and the number of bacteria was quantified, showing killing activity against MAH 4B2, but not *M*. *avium* 104. When MAH 4B2 was added to a HEp-2 monolayer covered with the mucin MUC2 in the presence of polymyxin B, the ability of the bacterium to enter the epithelial cell was significantly decreased compared with the wild-type *M*. *avium* 104 and with monolayer not containing polymyxin B (Table 7).

## 4. Discussion

The externally connected human host mucosal surface contains a layer or two of mucin, a glycoprotein intended to serve as a barrier to pathogens and other antigens [10, 11] and as a continuous cleaning mechanism for inhaled and ingested particles or microbes. Pathologic processes that impact the amount or fluidity of the mucin layer are associated with increase in the infections. Commonly observed conditions are chronic pulmonary diseases such as bronchiectasis, emphysema, and cystic fibrosis [23]. Those conditions course with the decrease in the mechanical defense of the lung mucosa, as well as the same for the intestinal mucosa.

Upon infection, cells producing mucin (mainly goblet cells) undergo hyperplasia and increased mucus secretion, proving protection against many different pathogens [24]. The layer of the mucin also participates in the tissue-related immune response, by incorporating antimicrobial peptides, enzymes such as lysozyme, and antibodies [11]. Recent publication also identified the innate lymphocytic cells (ILC 2) as an important trigger for mucin production in the sites connected with intestinal helminths [25].

Our investigation looked at the role of both intestinal and respiratory mucus in the mucosal defense against a nontuberculous mycobacteria, *M*. *avium*. The findings that the *M*. *avium* does not bind to either mucins (MUC2 and MUC5b), in contrast to *E*. *coli* and *P*. *aeruginosa*, suggest that *M*. *avium* crosses the mucin layer easily, explaining how the bacterium can attach to the mucosal surface without expressing a fimbria or long pili, which have been associated with the ability of another pathogen, *P*. *aeruginosa*, to bind and invade the lung airway mucosa [16].

Mucin protects against infection. A study reported by Bergstrom et al. and colleagues [14] investigating the role of pathogenic bacteria in lethal infectious colitis supports the concept that the mucin serves as a barrier to bacteria, preventing them from interacting with the mucosa surface. In fact, the work by Hashein et al. and colleagues [26] demonstrated that MUC2 deficiency resulted in increased susceptibility of mice to intestinal nematodes.

Pathogens interact with the mucus in different fashions. A common motility factor, flagella, is utilized by pathogenic bacteria such as *Vibrio cholera* to migrate through the mucus barrier. Similarly, another intestinal pathogen, *Salmonella*, appears to anchor itself to the mucus layer, using adhesions to promote colonization [27, 28].

Recent investigation showed that the airway mucus restricts *Neisseria meningitides* access to the nasopharyngeal epithelial cells, protecting the respiratory mucosa from the bacterium and the associated inflammatory response [29]. In that specific study, the presence of mucus on the cell surface prevented the inflammatory response from the epithelial cells, which may alter the interaction of the bacteria with the mucosal cells. In the current study, we showed that the presence of mucus on the monolayer did not alter the ability of *M*. *avium* strains to interact with respiratory epithelial cells, again confirming that the barrier between the bacterium and the mucosal cells is not able to interfere with the binding.

*M*. *avium* is an environmental bacterium that many times infects the host through contact with environment sources [30]. Previous studies have shown that the passage of the bacterium in environmental conditions, for example, affect the physical properties of the bacterial surface, making it more resistant to the pH of the stomach [22]. Since *M*. *avium* is encountered in water, and to infect the intestinal mucosal epithelial cells, it needs to cross the acid environment of the stomach, we exposed the bacterium to both conditions and examined its ability to bind to the mucin, which showed that those conditions had an effect on the ability of *M*. *avium* to interact with the mucin. In fact, the lack of influence was not surprising, since binding to the mucin would leave no deleterious effect on the ability to bind to mucosal cells.

All the evidence collected suggest that the bacterial surface had a major impact on the interaction with the mucin. *M*. *avium* surface, like other mycobacteria, contains glycoproteins, lipids, and carbohydrates [26]. In fact, by treating the bacterium with either a detergent (Tween) or trypsin caused significant alteration in the content of the cell wall, with change in the ability to bind to the mucin. The same kind of observation has been described in other microorganisms in the interaction with mucosal structures. All the work performed with the mucin and bacteria indicate that the role of mucin is to protect the mucosal surface from pathogens, and *M*. *avium* has evolved a surface, probably changed in a manner, that it does not bind to either MUC2 or MUC5b. Using a *M*. *avium* mutant that has an altered glycolipid structure on the cell wall also increased the binding of the bacterium to the mucin. Because *S*. *aureus* was also incapable to bind to the mucin, we can assume that although the surface lipids may inhibit the binding of *M*. *avium* to the mucin, other structures also present on the *S*. *aureus* surface may be involved in the inhibition. Many other pathogens have been described interacting with the mucin, such as *Campylobacter* and helminths [31, 32], either by changing gene expression or promoting mucin induction of T cell-simulation cytokines.

The lack of efficacy of the mucin to create a barrier between the pathogen and the mucosal cells was demonstrated using two different epithelial cell lines, HEp-2 and A549. The observation adds to a previous finding that showed *M*. *avium* resistance to the action of antimicrobial peptides [33]. Taking in consideration that both the intestinal and respiratory mucosa are covered by two layers of mucin, crossing the top layer will put the bacteria in contact with the bottom layer, which is rich in antibacterial peptides. In fact, in our studies, incubation with a surrogate of antimicrobial peptides significantly impaired the ability of the bacterium to invade epithelial cells in a mucin-containing monolayer. Therefore, the capability to prevent the action of antimicrobial peptides complement the bacterial phenotype.

In summary, the study has shown that *M*. *avium* is capable of bypassing the action of mucin, a natural barrier to pathogens, and interacts with epithelial cells on the mucosa, despite of the absence of a flagellum. How *M*. *avium* acquired that pathogenic characteristic is unknown at this point. Future work will explore this aspect of the pathogen, probably by screening for mutants that bind to the mucin, such as the 4B2 mutant in the work.

## Figures and Tables

**Table 1:** Binding of bacteria to the MUC2 mucin.

Bacteria	Adherence to MUC2 mucin at different concentrations		
	0.1 (%)	1 (%)	5 (%)
*M*. *avium* 104	0	0	0
*M*. *avium* 3388	0	0	0
*E*. *coli*	49.5 ± 0.6^*∗*^	51.0 ± 1.2^*∗*^	44.3 ± 0.8^*∗*^
*P*. *aeruginosa*	94.1 ± 0.4^*∗*^	92.6 ± 0.5^*∗*^	97.3 ± 0.3^*∗*^
*S*. *aureus*	0	0	0
*L*. *monocytogenes*	95.0 ± 0.2^*∗*^	96.2 ± 0.4^*∗*^	97.5 ± 0.2^*∗*^

Bacteria were prepared as described in Section 2. Agar/mucin and mucin control were used. The numbers represent the subtraction of adherence from the background (agar alone). The adherence of *E*. *coli*, *P*. *aeruginosa*, and *L*. *monocytogenes* was statistically significant compared to that of *M*. *avium* and *S*. *aureus* (*p* < 0.05).

**Table 2:** Binding of bacteria to mucin MUC5b.

Bacteria	Adherence to mucin MUC5b concentration (%)	
	1 (%)	5 (%)
*M*. *avium* 104	0	0
*M*. *avium* 3388	0	0
*E*. *coli*	59.0 ± 0.9^*∗*^	67.1 ± 1.6^*∗*^
*P*. *aeruginosa*	92.4 ± 0.3^*∗*^	93.8 ± 0.4^*∗*^
*L*. *monocytogenes*	94.3 ± 0.3^*∗*^	95.1 ± 0.3^*∗*^
*S*. *aureus*	0	0

Bacteria were prepared as described in Section 2. Agar mucin and agar control were used. The numbers represent the subtraction of adherence from the background (agar alone). The adherence of *E*. *coli*, *L*. *monocytogenes*, and *P*. *aeruginosa* was significant when compared to that of *M*. *avium* and *S*. *aureus* (*p* < 0.05).

**Table 3:** Effect of environmental conditions on the binding to mucin MUC2.

Bacteria	Binding to mucin (%)		
	H₂0 (%)	Acid pH (%)	7H9 (%)
*M*. *avium* 104	0	0	0
*M*. *avium* 3388	0	0	0

*M*. *avium* (strains 104 and 3388) was incubated in H_2_0 (overnight) or HBSS pH 4.0 (for 2 h); the number of bacteria was adjusted to 10^5^ organisms, and binding was performed to agar/mucin. *M*. *avium* collected in the 7H9 broth was used as a control.

**Table 4:** Invasion of epithelial cells by *M*. *avium* and *E*. *coli* in presence or absence of MUC2.

Bacteria	Invasion (%)			
	HEp-2		A549	
	None	5% mucin	None	5% mucin
*M*. *avium* 104	7.2 ± 0.3	7.0 ± 0.5	8.6 ± 0.4	8.3 ± 0.3
*M*. *avium* 3388	6.8 ± 0.4	6.6 ± 0.5	7.9 ± 2	7.6 ± 0.7
*E*. *coli*	27 ± 5	16.7 ± 3 (^*∗*^)	20 ± 0.9	11.0 ± 0.4 (^*∗*^)

(^*∗*^) *p* < 0.05 compared with the invasion without the presence of mucin. Bacteria were prepared as described in Section 2. Epithelial cells, 90% confluent, were covered with 5% mucin, and bacterial invasion was determined by incubating bacteria with culture cells (with or without mucin) and quantifying the number of intracellular bacteria (percent of invasion). Initial inoculum of 2 × 10^5^ for *M*. *avium* 104, 2.4 × 10^5^ for *M*. *avium* 3388, and 3.3 × 10^5^ for *E*. *coli* was used.

**Table 5:** Effect of Tween-20 and trypsin treatment on the binding to the MUC2 mucin.

Bacteria	% binding to MUC2 mucin		
	Control (7H9)	Tween-20	Trypsin
*M*. *avium* 104	0	47 ± 6^*∗*^	38.1 ± 4^*∗*^
*M*. *avium* 3388	0	54 ± 6^*∗*^	48.4 ± 5^*∗*^

*p* < 0.05 compared to *M*. *avium* incubated in the 7H9 broth. *M*. *avium* strains were treated with Tween-20 or trypsin for 3 h at 37°C, washed, and incubated with 5% MUC2 mucin. The numbers represent the percent of binding to the mucin.

**Table 6:** *M*. *avium* 104, A5, and 4B2 mutant binding to the mucin.

Bacteria	Binding	
	% bacteria to MUC2 mucin	% bacteria to MUC5b mucin
*M*. *avium* 104	0	0
*M*. *avium* A5	0	0
*M*. *avium* 4B2	32 ± 3^*∗*^	36 ± 8^*∗*^

Results demonstrated that the cell wall-associate mutant has increased ability to interact with MUC2 and MUC5b mucins. ^*∗*^*p* < 0.05 compared to the binding of the wild-type bacteria. *M*. *avium* 4B2 has been described previously, and it is deficient in colonization of the intestinal and respiratory mucosa. The mutant and wild-type were inhabited in a surface covered by MUC2 or MUC5b.

**Table 7:** Effect of polymyxin B on the invasion of HEp-2 cells in presence of the MUC2 mucin.

Bacteria	Polymyxin B	Invasion HEp-2	Invasion HEp-2 + polymyxin B
*M*. avium 104	4.5 ± 0.5 × 105	8.3 ± 0.4 × 104	8.0 ± 0.5 × 104
MAH 4B2	2.5 ± 0.6 × 103^*∗*^	3.2 ± 0.5 × 103^*∗*^	6.4 ± 0.4 × 102^*∗*&^

Inoculum was 4.5 × 10^5^ for *M*. *avium* and 3 × 10^5^ for MAH 4B2. Bacteria were incubated with polymyxin for 4 hours, and the diluted and plated bacteria were placed on the monolayers with or without polymyxin for 2 h; then extracellular bacteria were removed, and HEp-2 cells were lysed. The lysate was plated for quantification of colony forming units. ^*∗*^*p* < 0.5 for the comparison between *M*. *avium* 104 and 4B2. &*p* < 0.5 for the comparison between MAH 4B2 invasion of HEp-2 cells with and without polymyxin B.

## Data Availability

All the data included in the manuscript are available any time upon request.
